# The utility of measuring the apparent diffusion coefficient for peritumoral zone in assessing infiltration depth of endometrial cancer

**DOI:** 10.1186/s40644-018-0156-6

**Published:** 2018-07-03

**Authors:** Lei Deng, Qiu-ping Wang, Rui Yan, Xiao-yi Duan, Lu Bai, Nan Yu, You-min Guo, Quan-xin Yang

**Affiliations:** 1 Department of Radiology, the First Affiliated Hospital, Xi’an Jiaotong University Xi’an, #277, Yanta West Road, Xi’an, 710061 Shaanxi China; 2 Department of Radiology, the Northwest Women and Children Hospital, #1616, Yanxiang Road, Xi’an, 710054 Shaanxi China; 3 Department of Nuclear Medicine, the First Affiliated Hospital, Xi’an Jiaotong University Xi’an, #277, Yanta West Road, Xi’an, 710061 Shaanxi China; 4 Department of Radiology, The Affiliated Hospital of Shaanxi University of traditional Chinese Medicine, #2. Wei Yang West Road, Xian Yang, 712000 Shaanxi China

**Keywords:** Diagnostic imaging, Diffusion, Endometrial carcinoma, Endometrial neoplasm, Magnetic resonance imaging

## Abstract

**Background:**

The invasion depth of endometrial cancer is one of the most important prognosis factors. The aim of the current study was to investigate the diagnostic value of the apparent diffusion coefficient (ADC) of the peritumoral zone for assessing the infiltration depth of endometrial cancer.

**Methods:**

An institutional review board approved this prospective study, and all study participants provided informed consent. A total of 58 patients (mean age 54 ± 8.3 years, range 34–69 years) with endometrial cancer were prospectively enrolled. Two radiologists assessed all preoperative magnetic resonance images with T1, T2, and diffusion-weighted imaging, and determined the location of the deepest invasion of the tumor. The peritumoral zone was defined as a 5-mm-thick zone surrounding and adjacent to the cancerous endometrium. The mean ADC (ADCm) values of the tumor and the peritumoral zone were measured. Sensitivity, specificity, positive and negative predictive values, and the area under the receiver operating characteristic curve (Az) were calculated for visual inspection, and an ADC cutoff value for the peri-endometrial zone was determined for predicting the myometrial invasion depth.

**Results:**

The ADCm values of tumors and peritumoral zones were 0.83 × 10^− 3^ mm^2^/sec and 1.06 × 10^− 3^ mm^2^/sec, respectively. There was no significant difference between the ADCm values of the tumors in the superficial and deep myometrial invasion groups (P > 0.05). However, the ADCm value at the peritumoral zone in the deep myometrial invasion group (1.23 × 10^− 3^ mm^2^/sec) significantly differed from that in the superficial myometrial invasion group (0.99 × 10^− 3^ mm^2^/sec) (*p* = 0.005). In assessments of deep myometrial invasion, the sensitivity, specificity, negative predictive value, and positive predictive value were 0.58, 0.93, 0.84, and 0.77, respectively, for the ADCm cutoff value of the peritumoral zone, and 0.71, 0.80, 0.87, and 0.60. respectively, for visual inspection. The accuracy of myometrial invasion depth assessment using the ADCm cutoff value and visual inspection were 83 and 78%, respectively. The Az for both was 0.76.

**Conclusion:**

ADCm at the peritumoral zone can predict deep myometrial invasion of endometrial cancer. This value can therefore enhance confidence in preoperative endometrial cancer evaluation, and when tailoring surgical approaches.

**Electronic supplementary material:**

The online version of this article (10.1186/s40644-018-0156-6) contains supplementary material, which is available to authorized users.

## Background

Endometrial cancer is the sixth most common malignant disorder in women worldwide [1]. Its prognosis depends on multiple factors,with the depth of myometrial invasion being one of most important [2]. This depth may be used as a surrogate marker to determine possible lymphovascular space invasion and the risk of lymph node metastases [3, 4]. The prevalence of lymph node metastases increases from 3% with superficial myometrial invasion to 46% with deep myometrial invasion [5], and the recurrence risk was reportedly intermediate to moderately high in patients with deep myometrial invasion [6]. Therefore, accurate preoperative delineation of the myometrial invasiveness of endometrial cancer is essential.

Magnetic resonance imaging (MRI) is recommended for the management and preoperative staging of endometrial cancer [7]. Recently, diffusion-weighted (DW) imaging has been introduced to better evaluate tissue composition in gynecologic tumors [8]. DW images can qualitatively analyze the myometrial invasion depth of endometrial cancer [9, 10], especially in combination with T2WI [11]. Apparent diffusion coefficient (ADC) values allow normal endometrium or benign lesions to be differentiated from endometrial carcinoma [12, 13]; however, they could not quantitatively diagnose the myometrial infiltration depth of endometrial cancer [13, 14]. According to FIGO 2009, a tumor that invades ≥50% of the myometrium is defined as deep myometrium infiltration of endometrial cancer [15], whereas superficial myometrial invasion is defined as tumor invasion < 50% of the myometrium invasion depth. The integration of the junctional zone is very important for the assessment of myometrium infiltration depth [16]. However, in the presence of pitfalls such as a loss of junctional zone definition, poor tumor to myometrium contrast, myometrial compression by polypoid tumor, leiomyomas, and adenomyosis, morphologic inspection are challenging for the accurate assessment of myometrial invasion depth [17]. As the previously studies reported, in a normal uterus, the ADC value of junctional zone was the lowest among the three layers and the highest in the outer myometrium [18, 19]. The most important prognostic factor is the variation of invasion depth with different degrees of integration of the junctional zone, as mentioned above. Hence, a change in diffusion may also be present in the peritumoral zone of endometrial cancer.

The purpose of this study was to explore the diagnostic value of the ADC value of the peritumoral zone for predicting the myometrial invasion depth of endometrial cancer in comparison with the ADC value of the tumor.

## Methods

### Study population

Our institutional review board approved this prospective study, and all study participants provided informed consent. All patients were histopathologically confirmed to have primary untreated endometrial cancer via fractional dilatation and curettage or biopsy. Patients were excluded if they had (a) any contraindications for MRI (such as a cardiac pacemaker or defibrillator, insulin pump, aneurysm clip, implanted neural stimulator, cochlear implant, or metal shrapnel or bullet); (b) pelvic or hip metal prostheses; (c) not provided informed consent; (d) any contraindications for surgery; or (e) unavailable postoperative histological reports.

Between April 2012 and January 2014, based on surgery and pathology, a total of 58 consecutive patients (mean age 54 ± 8.3 years, range 34–69 years) were enrolled. All the patients underwent pelvic MRI as part of their initial staging before surgery.

### Imaging protocol

The MRI was performed with a 3.0-T MRI unit (Signa HDx 3.0 T, GE Medical Systems, GE Healthcare, Waukesha, Wis, USA) with an 8US TORSOPA coil. All subjects had fasted for 6 h and were trained to hold their breaths at the end of expiration before scanning. For all examinations, patients were placed in the supine position and had a partially filled bladder. T1-weighted, T2-weighted, and DW images of the pelvis were acquired. Fast spin-echo T2-weighted images were initially obtained in the sagittal, axial, and coronal planes with the following parameters: repetition time (TR)/echo time (TE) 3680–6240 ms/85–89 ms, field of view 30–35 cm, number of acquired signals 2, section thickness 5 mm, and bandwidth 35.71–83.33 KHz. Following this sequence, axial fast spin-echo T1-weighted images were acquired with the following parameters: number of acquired signals 2, section thickness 5 mm, and bandwidth 50 KHz.

Axial oblique DW imaging (oblique to the corpus) of the pelvis was performed using the single-shot echo-planar technique with fat suppression (TR/TE 5000 ms/67.6 ms, matrix 128 × 128, field of view 35 × 35 cm, number of acquired signals 4, section thickness 5 mm, and *b* values 0 and 1000 s/mm^2^). The array spatial sensitivity encoding technique was used as the parallel imaging technique during DW image scanning. The ADC map of each DW image was produced with a GE Advantage Windows (AW) 4.4 Workstation.

### Imaging analysis

**Fig. 1:**
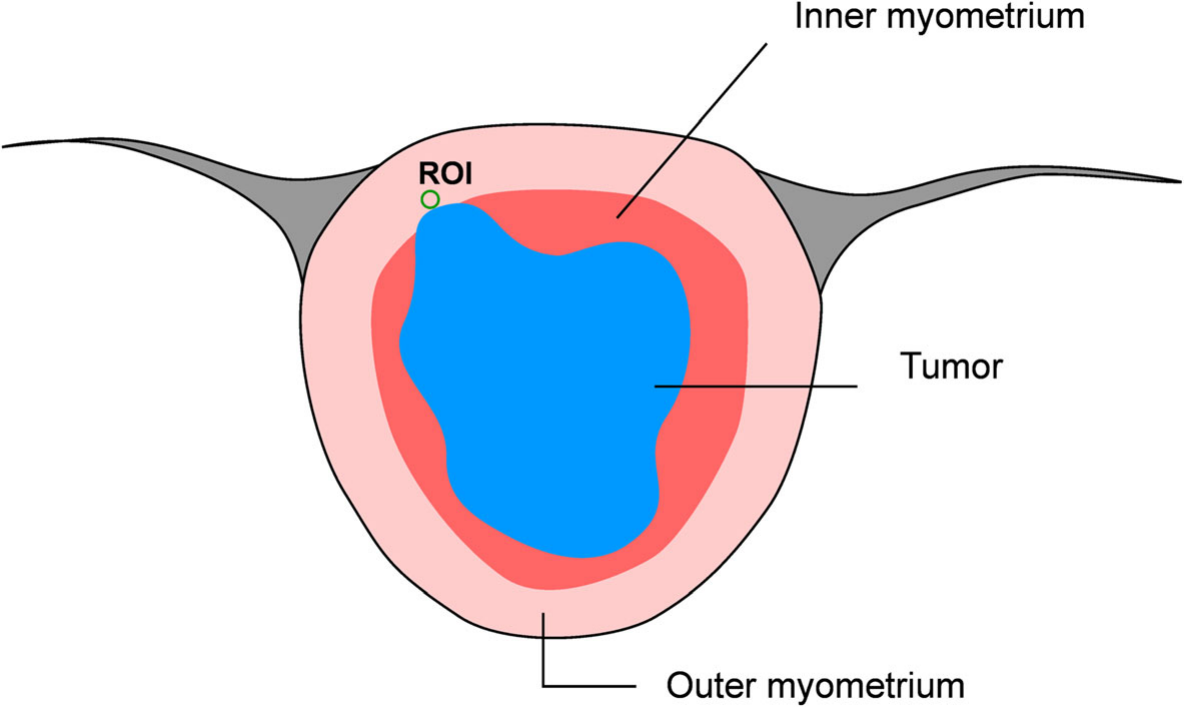
The pictorial illustration of ROI placement on peritumoral zone of endometrial cancer. The tumor area is in blue, and the inner & outer myometrium is in red&pink. An elliptical region of interest (ROI) was drawn along the margin of deepest invasion of the tumor (i.e., peritumoral zone)

**Fig. 2:**
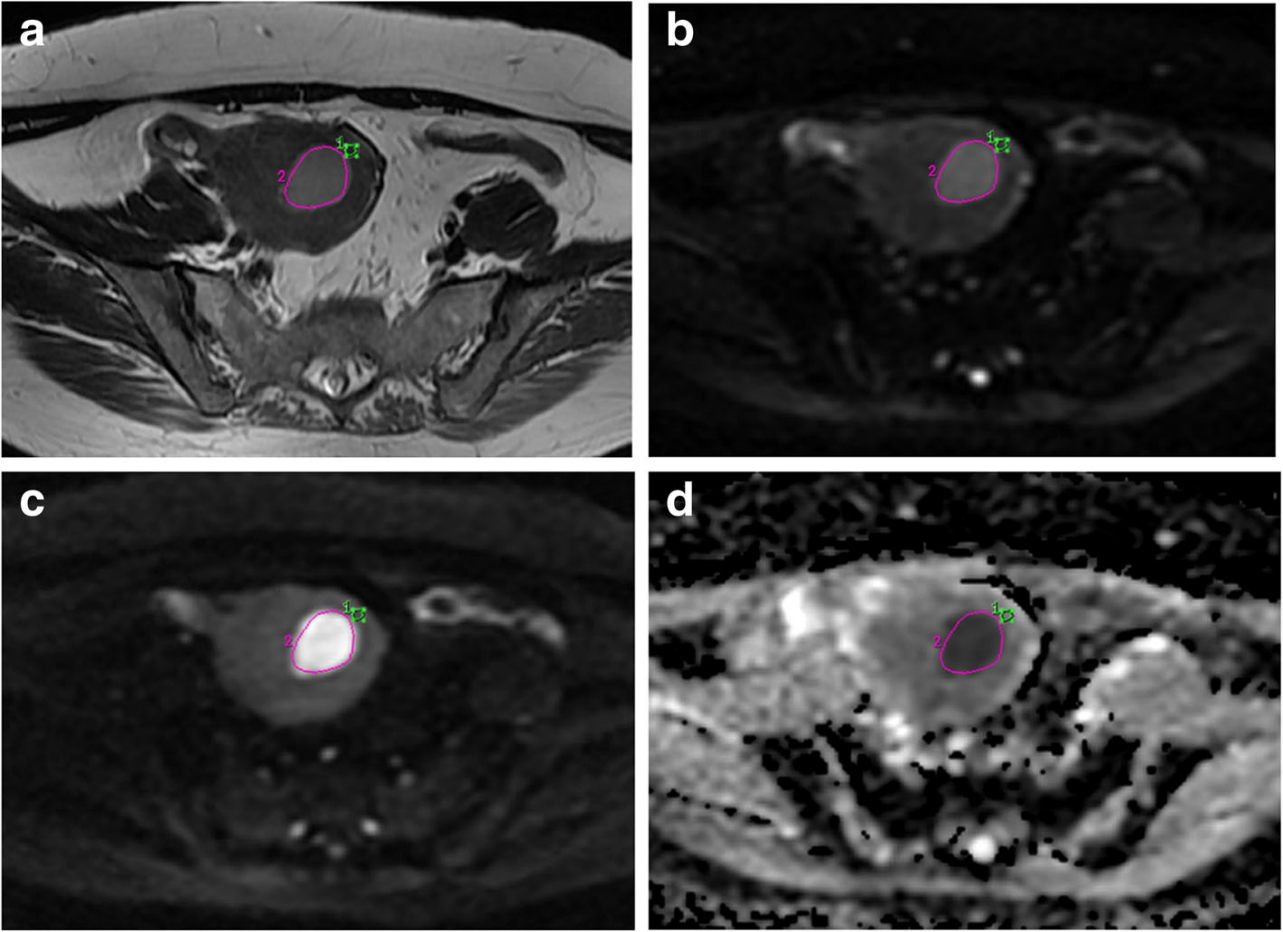
Endometrial adenocarcinoma with superficial myometrial invasion in a 43-year-old woman. An elliptical region of interest (ROI) was placed on peritumoral zone (ROI_1_ with green color), which was assessed subjectively on a cross-section of the T2-weighted image obtained by echo planar imaging (*b* = 0 s/mm^2^). In addition, a freehand ROI (ROI_2_ with pink color) was placed on the tumor which contained the largest endometrial cancerous area, avoiding artifacts from the neoplastic/non- neoplastic interface and visible lesions or vascular structures in the myometrium. **a** T2-weighted image; **b** diffusion-weighted magnetic resonance image (*b* = 0 s/ mm^2^); **c** diffusion-weighted magnetic resonance image (*b* = 1000 s/mm^2^); **d** apparent diffusion coefficient map

All MR sequences were randomized in order and viewed by two radiologists with 10 and 8 years of experience in gynecologic radiology, who were blinded to the histopathological findings and patients’ names, but were aware that the patients had been diagnosed with endometrial cancer. Disagreements were resolved by consensus. For visual inspection, the readers evaluated the standard anatomic sequences (T1-and T2-weighted imaging) as well as the DW images for the depth of myometrial invasion, which was scored as ‘superficial’ if the tumor invaded up to 50% of the myometrial thickness and ‘deep’ if the tumor extended beyond 50% of the myometrium thickness. Tumor maximal diameter (as tumor size) [20–22] was calculated on multiple sequences, and the largest value was recorded. Quantitative analysis of DW images was performed using ADC maps which were generated on the scanner console using the *b* = 1000 s/mm^2^ and *b* = 0 s/mm^2 ^images. Regions of interest (ROIs) were applied to tumors and peritumoral zones. The peritumoral zone was defined as a 5-mm-thick zone surrounding and adjacent to the cancerous endometrium [23]. The radiologists reviewed the T2 and DW images and determined the location of the deepest invasion of the tumor. An elliptical ROI (mean area, 20mm^2^) was then drawn along the deepest invasion margin of the lesion for measuring the ADC of the peritumoral zone. The pictorial illustration of ROI placement on peritumoral zone is depicted in Fig. 1. For measuring the ADC of the tumor, ROIs were applied to the tumor that contained the largest endometrial cancerous area, avoiding artifacts from the neoplastic/non-neoplastic interface and visible lesions or vascular structures in the myometrium. The ROI setting was on the cross-section of the T2-weighted image obtained via echo planar imaging (*b* = 0 s/mm^2^), and it was manually copied to the corresponding ADC map, whereupon ADC values were automatically calculated (Fig. 2). For quality control, the placements of ROIs were determined by the two radiologists. Disagreements were resolved by consensus. The measurement was repeated three times and the interval between the measurements was 1 week. The final data recorded was ADCm (mean ADC) value averaged from the three measurements by the two radiologists.

### Histopathological analysis

All endometrial cancer patients underwent a total abdominal hysterectomy and bilateral salpingo-oophorectomy, including 20 patients who underwent pelvic lymphadenectomy simultaneously. After resection, the uterus was cut into 5 mm-thick axial sections for evaluation of myometrial invasion depth, which was performed the same way as the MRI interpretation. A pathologist with 15 years of experience in gynecologic disease who was blinded to the imaging results assessed FIGO stage, histological type, tumor grade (G1, well differentiated; G2, moderately differentiated; and G3, poorly differentiated), and depth of myometrial invasion (superficial myometrial invasion, the tumor invades < 50% of the myometrium; deep myometrial invasion, the tumor invades ≥50% of the myometrium).

### Statistical analysis

The ADCm values of the superficial and deep myometrial invasion of the tumor or peritumoral zone were compared using the independent sample *t*-test; two-tailed *p* values of < 0.05 were considered statistically significant. The cutoff ADCm value of the peritumoral zone in endometrial cancer was obtained by drawing a receiver operating characteristic (ROC) curve. In order to maximize both of sensitivity and specificity, we applied the Youden’s index (Youden’s index = Sensitivity+Specificity-1). We chose the point closest to the upper left corner of the curve as a cutoff, where the Youden’s index was maximal. The cutoff ADCm value and visual inspection were used as the diagnostic indexes to evaluate deep myometrial invasion. The sensitivity, specificity, positive predictive value (PPV), and negative predictive value (NPV) of the ADCm cutoff and visual inspection were calculated and represented with 95% confidence intervals. All statistical analyses were performed by IBM SPSS statistical software, version 19.0. The ROC curve was drawn using Stata/SE 12.0 for windows. The pictorial illustration of the ROI placement was drawn by FREEHAND, version 11.0.2.

## Results

### Histopathological findings

The intervals between MRI examination and surgery were 0–21 days (mean 4 days). Of the 58 patients with endometrial cancer, postoperative histological assessment revealed endometrioid adenocarcinoma in 43, adenosquamous carcinoma in 11, mixed endometrioid/serous papillary carcinoma in 2, and mixed endometrioid/mucinous papillary carcinoma in 2. The tumor was confined to the endometrium or involved the inner half of the myometrium (superficial myometrial invasion) in 41 cases, and involved the outer half of the myometrium (deep myometrial invasion) in the remaining 17 cases. The relevant histopathological findings are shown in Table 1.

**Table 1:** Patients’ surgical and pathological findings

	Variable	Data
Myometrial invasion	superficial	41
	deep	17
	endometrioid	43
Histological type	adenosquamous	11
	mixed endometrioid/mucinous papillary	2
	mixed endometrioid/serous papillary	2
Histological grade	1	13
	2	36
	3	9

*FIGO* International federation of gynecology and obstetrics

### Quantitative analysis

**Fig. 3:**
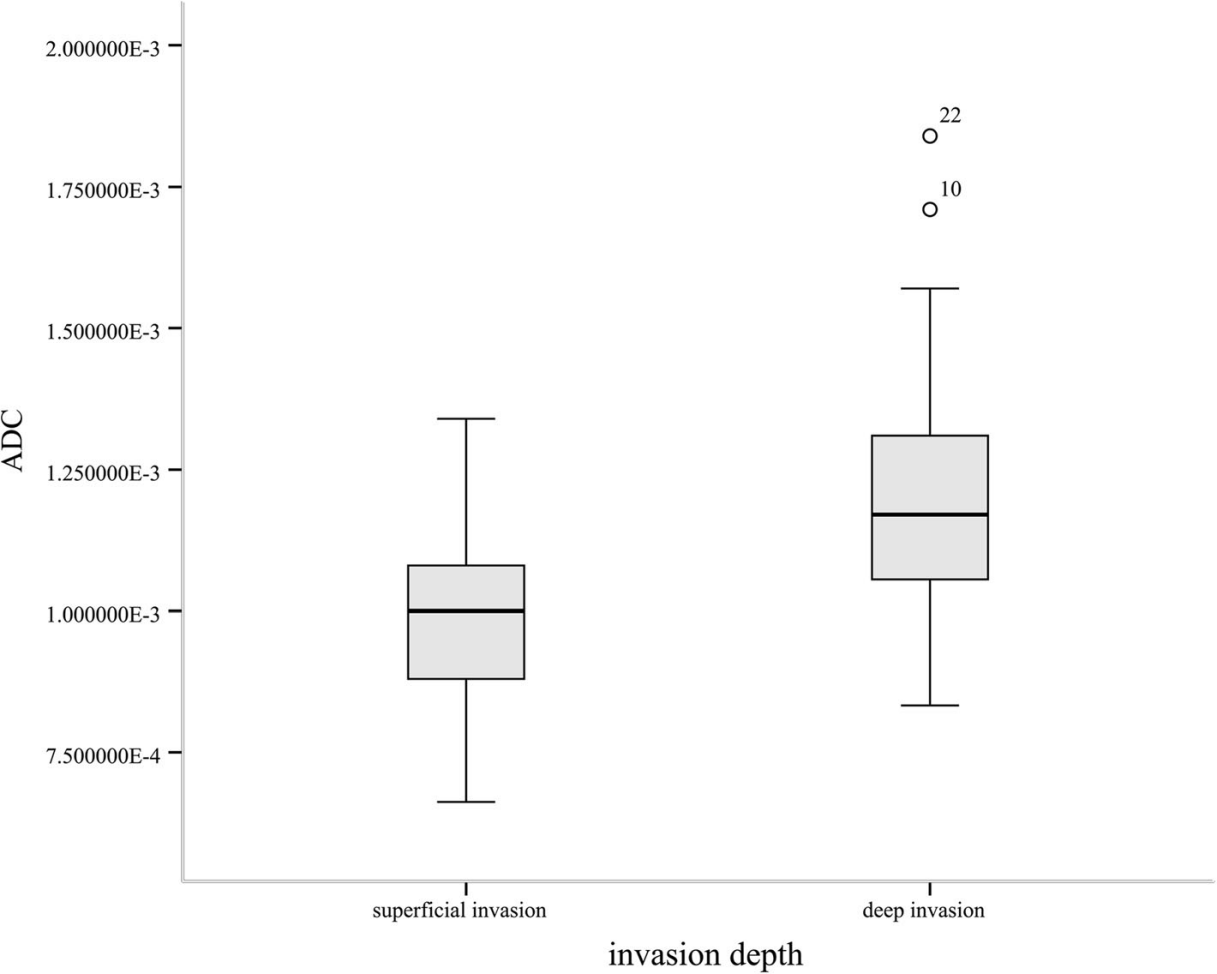
It is a box and whisker plot which is the correlation between ADC value of peritumoral zone and histological depth of invasion

Of the 58 endometrial cancers, the mean tumor size was 3.9 ± 1.9 cm. The ADCm values of tumor and the peritumoral zone were (0.83 ± 0.11) × 10^− 3^ mm^2^/sec and (1.06 ± 0.22) × 10^− 3^ mm^2^/sec, respectively. There was no significant difference between the ADCm values of tumor in the superficial and deep myometrial invasion groups (superficial invasion, 0.84 × 10^—3^ mm^2^/sec and deep invasion, 0.82 × 10^—3^ mm^2^/sec; *p* >  0.05). The ADCm value at the peritumoral zone of the deep myometrial invasion and that of the superficial myometrial invasion were 1.23 × 10^—3^ mm^2^/sec and 0.99 × 10^—3^ mm^2^/sec, respectively, which was a significant difference (*p* = 0.005) (Table 2 and Fig. 3).

**Table 2:** Apparent diffusion coefficient values for different depth of myometrial invasion (× 10^− 3^ mm^2^/sec)

	Peritumoral zone		Tumor	
	ADC	p	ADC	p
Superficial	0.99 ± 0.15	0.005*	0.84 ± 0.10	> 0.05
Deep	1.23 ± 0.27		0.82 ± 0.14	

*ADC* Apparent diffusion coefficient

^*^p < 0.05 was considered a statistically significant difference

### Diagnostic performance of the ADC cutoff value of the peritumoral zone and the visual inspection

**Fig. 4:**
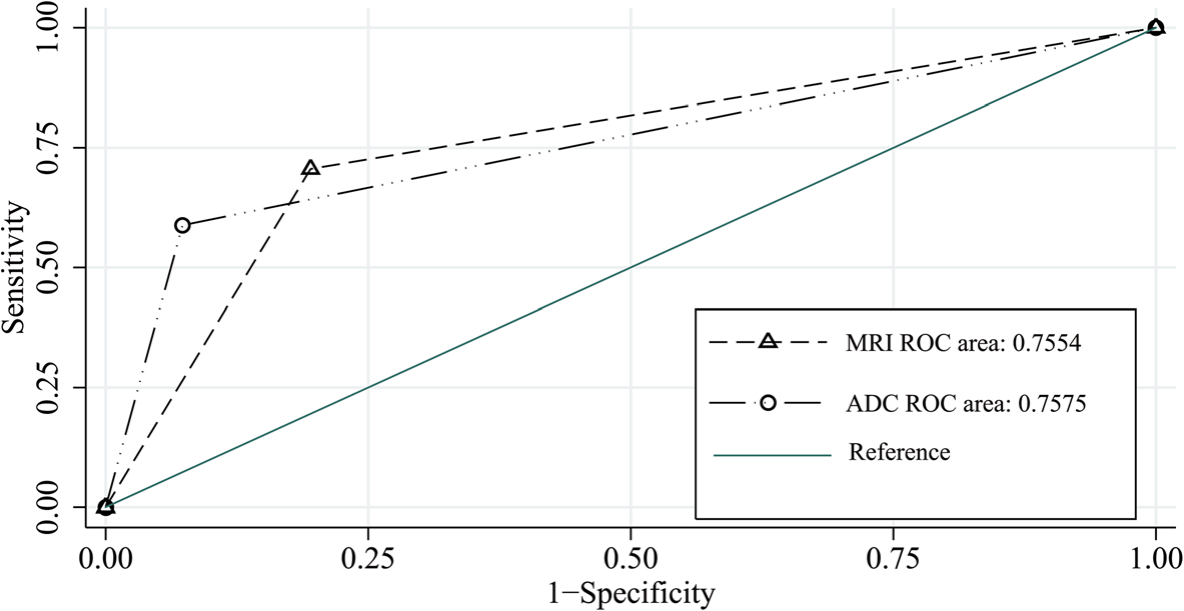
Az values of the two methods obtained from ROC

The diagnostic performances of the two methods for assessing deep myometrial involvement are summarized in Table 3. The ADC cutoff value of the peritumoral zone for assessing deep myometrial invasion was 1.17 × 10^− 3^ mm^2^/sec. An additional figure file shows this in more detail [see Additional file 1]. For assessing deep myometrial invasion of endometrial cancer, the specificity for the ADCm cutoff value of the peritumoral zone (0.93) was higher than for visual inspection (0.80), as were the PPVs (ADCm, 0.77; vs. visual inspection, 0.60). The areas under the ROC curve (Az) were 0.76 for both methods, but the diagnostic accuracy for the ADCm cutoff value (83%) was higher than for visual inspection. The ROC curves are depicted in Fig. 4.

**Table 3:** Diagnostic performance of deep myometrial invasion assessment by ADC cutoff value and visual inspection of peritumoral zone

Method	Findings				Acc	Az	Sensitivity (95% CI)	Specificity (95% CI)	NPV (95% CI)	PPV (95% CI)
	TP	FP	FN	TN						
ADC cutoff	10	3	7	38	0.83	0.76	0.59	0.93	0.84	0.77
							(0.33–0.81)	(0.79–0.98)	(0.70–0.93)	(0.46–0.94)
Visual inspection	12	8	5	33	0.78	0.76	0.71	0.80	0.87	0.60
							(0.44–0.89)	(0.65–0.91)	(0.71–0.95)	(0.36–0.80)

Data are means and numbers in parentheses are 95% confidence intervals

*ADC* Apparent diffusion coefficient, *Az* Area under the receiver operating characteristic curve, *Acc* Accuracy, *NPV* Negative predictive value, *PPV* Positive predictive value, *CI* Confidence interval, *TP* True-positive, *FP* False-positive, *FN* False-negative, *TN* True-negative

## Discussion

Our results suggested that the ADCm of a tumor could not differentiate deep myometrial invasion from superficial myometrial invasion in endometrial cancer, which is concordant with previous researches [13, 14]. However, the ADCm of the peritumoral zone in the deep myometrial invasion group differed significantly from that of the superficial myometrial invasion group, and was therefore potentially useful for ruling out deep myometrial invasion. Moreover, it was more accurate than visual inspection for assessing deep myometrial invasion, and so could be used as a quantitative MRI tool for helping assess deep myometrial invasion of endometrial cancer.

DW imaging is a functional technique that provides information about water mobility, tissue cellularity, and the integrity of the cellular membrane. In biological tissues, water mobility, i.e., Brownian motion, is restricted via interaction with cell membranes and macromolecules at a microscopic level. In addition to providing essential qualitative information regarding the diffusivity of water molecules in a given tissue, DW imaging enables quantitative information to be obtained with the use of ADC maps [24]. Calculating the ADC can provide quantitative analysis of Brownian motion. The higher the signal of a region in a DW image, the lower the ADC values are, indicating thicker tissues with more densely populated cells [25].

In this study, the peritumoral zone was defined as a 5-mm-thick zone surrounding and adjacent to the cancerous endometrium. In patients with normal endometrium, it is the junctional zone of the uterus. Three distinct layers can be visualized via T2-weighted MRI in a normal uterus: a high signal intensity layer corresponding to the endometrial stripe, an inner low signal intensity layer that is adjacent to the basal endometrium (the junctional zone or subendometrial layer), and an outer medium signal intensity subserosal zone or outer myometrium [26]. The junctional zone has increased nuclear area, decreased extracellular matrix, and lower water content in comparison with the outer myometrium. In addition, junctional zone myocytes are thought to express different extracellular matrix components [27, 28]. These features not only shorten the T2 but also restrict diffusion, which gives rise to a low signal zone on the ADC map and the lowest ADC value of this region in the normal uterus [18]. Previous studies showed that in the normal uterus, the ADC value of the junctional zone was the lowest among the three layers and that of the outer myometrium was the highest [18, 19]**.** In deep myometrium infiltration of endometrial cancer, the tumor invades ≥50% of the myometrium and thus appears as a complete disruption of the junctional zone. When this happens, the peritumoral zone actually includes a majority of the outer myometrium and a small amount of cancerous tissue, which is indicated by a higher signal zone on T2-weighted imaging and ADC maps in comparison with the normal junctional zone. In contrast, in superficial myometrial invasion (< 50% of myometrium invasion depth), the peritumoral zone consists of partial junctional zone, partial outer myometrium, and a small amount of cancerous tissue which exhibit a lower signal in comparison with deep myometrial invasion on T2-weighted imaging and ADC maps. Accordingly, there should be a restricted diffusion difference between deep and superficial myometrial invasion. This was confirmed by our result showing that the ADCm of peritumoral zone of deep myometrial invasion (1.23 × 10^− 3^ mm^2^/sec) was significantly greater than that of superficial myometrial invasion (0.99 × 10^− 3^ mm^2^/sec) (*p* = 0.005). Thus, the ADCm value of the peritumoral zone may provide useful information for differentiating deep myometrial invasion from superficial myometrial invasion in endometrial cancer.

In the current study, in endometrial cancer patients, the ADCm values of the tumor exhibiting deep myometrial invasion and superficial myometrial invasion did not differ significantly. This finding is concordant with results previously reported by Lin et al. [14] and Rechichi et al. [13]. A possible explanation for this finding is that cellular density and medium interstice are the main factors affecting Brownian motion. A tissue with high cellular density and medium interstice, such as neoplastic tissue, corresponds to high signal in DW imaging. Conversely, tissue with lower signal in DW imaging (i.e., normal issue) corresponds to a region with a higher ADC value [24]. Notably however, other important features of tumor cells such as nuclear atypia cannot be assessed by DW imaging [29]. That is, the ADC value alone is not sufficient for ascertaining the invasiveness of a tumor.

The current study had some limitations. One was the small size of the deep myometrial invasion group. The deep infiltration group included 17 patients (29.3% of the entire study group), which might have biased the sensitivity, specificity, PPV, and NPV of the two assessment methods such that they did not reach statistical significance. Moreover, the study lacked objective assessments to determine the location of the deepest myometrial invasion where an ROI should be set.

## Conclusion

The ADC value obtained at the peritumoral zone can predict deep myometrial invasion of endometrial cancer. This value could therefore enhance confidence in the preoperative evaluation of endometrial cancer, and be useful when tailoring the surgical approach.

## Additional file

Additional file 1: A ROC curve of the ADCm value of the periturmoral zone in endometrial cancer. The cutoff ADCm value of the peritumoral zone was obtained from the curve. We chose the point closest to the upper left corner of the curve as a cutoff, where the Youden’s index (Youden’s index = Sensitivity+Specificity-1) was maximal. (TIF 1474 kb)

## Abbreviations

ADC: Apparent diffusion coefficient
ADCm: Mean apparent diffusion coefficient
DW: Diffusion-weighted
FIGO: Federation of Gynecology and Obstetrics
MRI: Magnetic resonance imaging
NPV: Negative predictive value
PPV: Positive predictive value
ROC: Receiver operating characteristic
ROI: Region of interest
TE: Echo time
TR: Repetition time
